# Evolution Characteristics of the Network Core in the Facebook

**DOI:** 10.1371/journal.pone.0104028

**Published:** 2014-08-28

**Authors:** Jian-Guo Liu, Zhuo-Ming Ren, Qiang Guo, Duan-Bing Chen

**Affiliations:** 1 Research Center of Complex Systems Science, University of Shanghai for Science and Technology, Shanghai, People's Republic of China; 2 Department of Physics, University of Fribourg, Chemin du Musée 3, Fribourg, Switzerland; 3 Web Sciences Center, University of Electronic Science and Technology of China, Chengdu, People's Republic of China; Wake Forest School of Medicine, United States of America

## Abstract

Statistical properties of the static networks have been extensively studied. However, online social networks are evolving dynamically, understanding the evolving characteristics of the core is one of major concerns in online social networks. In this paper, we empirically investigate the evolving characteristics of the Facebook core. Firstly, we separate the Facebook-link(FL) and Facebook-wall(FW) datasets into 28 snapshots in terms of timestamps. By employing the *k*-core decomposition method to identify the core of each snapshot, we find that the core sizes of the FL and FW networks approximately contain about 672 and 373 nodes regardless of the exponential growth of the network sizes. Secondly, we analyze evolving topological properties of the core, including the *k*-core value, assortative coefficient, clustering coefficient and the average shortest path length. Empirical results show that nodes in the core are getting more interconnected in the evolving process. Thirdly, we investigate the life span of nodes belonging to the core. More than 50% nodes stay in the core for more than one year, and 19% nodes always stay in the core from the first snapshot. Finally, we analyze the connections between the core and the whole network, and find that nodes belonging to the core prefer to connect nodes with high *k*-core values, rather than the high degrees ones. This work could provide new insights into the online social network analysis.

## Introduction

Online social networks are organized around participating users who create interactions with whom they associate [1]–[3]. As online social networks are gaining more attentions, more than a billion people have been integrated to make friends, communicate with friends, share interests, spread ideas and so on [4], [5]. An in-depth investigation of the evolving network core is very important for deeply understanding the evolving characteristics of online social networks [6], [7], where the core could be identified by the k-core decomposition method [8]. Carmi *et al.* [9], Zhang *et al.* [10], and Orsini *et al.* [11] investigated the topological properties of the internet at the autonomous system level, and found that the internet core was a small and well connected subgroup, specifically its size was approximately stable over time. Kitsak *et al.* [12] employed the k-core decomposition method to identify the most influential spreaders which is defined as the nodes with the highest *k*-core value(i.e. core). Miorandi *et al.* [13], Ren *et al.* [14] extended the *k*-core decomposition method to identify the node spreading influence in networks, and Garas *et al.* [15] presented a generalized method for calculating the *k*-core structure of weighted networks. These works have similar conclusions that nodes belonging to the core are the most influential spreaders. Regarding to the internet network analysis, little attention has been paid to the core properties of the online social networks. In this paper, we empirically analyze the evolution characteristics of the Facebook's core, and the statistical results indicate that (1) The core sizes of the Facebook-link(FL) and Facebook-wall(FW) networks are approximately stable around 672 nodes and 373 nodes respectively. (2) Nodes belonging to the core get more interconnected, and their *k*-core values increase correspondingly. (3) The life span analysis of the nodes belonging to the core reveals that more than 50% nodes stay in the core for more than one year, and 19% nodes always stay in the core from the first snapshot. (4) The nodes in the core prefer to connect to high *k*-core nodes, regardless of the high degree ones.

## Materials and Methods

### Datasets

The Facebook datasets [16] are investigated in this paper, which consist of two different components. The first one is the Facebook-link(FL) that spans from September 5, 2006 to January 22, 2009. The timestamp of each link indicates the time when one pair of users become friends. The other one is the Facebook-wall(FW) that spans from September 14, 2004 to January 22, 2009. It should be noticed that a user can post comments on his/her friends' walls, and these comments can be seen by visitors. In this paper, we treat these interactions as undirect links. The information of each link in the FW network consists three parts: The wall owner, the user who posted and the corresponding posted time. In order to compare the evolution characteristics of the network core between the FL and FW networks, the period investigated in this paper is set from September 2006 to December 2008.

Firstly, we separate the FL network into pieces with the interval of one month. Since approximately 41% timestamps of links could not be determined, we set this kind of links as the initial network 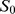. The first piece 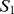 is set from September 1, 2006 to September 30, 2006. The second one 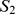 is set from October 1, 2006 to October 31, 2006, and the last one 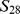 is set from December 1, 2008 to December 31, 2008. Based on the initial network 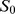 and 28 pieces, we can construct 28 corresponding snapshots. The first snapshot is defined by merging 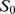 and 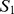. The second one consists of 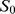, 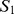 and 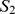. The last one consists of the initial network 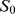 and all pieces 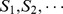 and 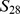. Similarly, the FW network can also be separated into 28 snapshots. It is emphasized that in the FW network, the initial network 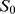 corresponds to September 14, 2004 to August 31, 2006.

### Methods

**Figure 1:**
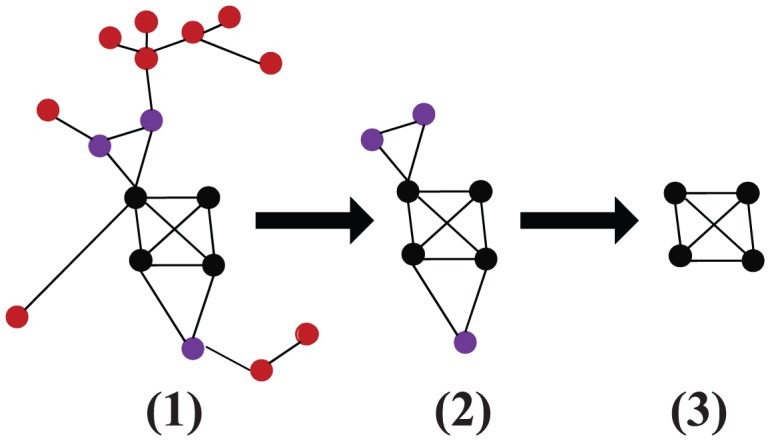
(Color online) Illustration of *k*-core decomposition. (1) is 1-core, (2) is 2-core, (3) is 3-core, i.e. core of the network.

Identifying the network core has been extensively investigated [9]–[13], [ 15], [ 17]–[19]. For example, the core might be defined as the set of all nodes with degree higher than some threshold. But this method requires setting a free parameter, the degree threshold. Other methods [18] like k-clique, k-clan and some improved methods based k-core methods like k-dense [11], Medusa-model [9] are used to identify the AS network core. In this paper, we focus on investigating the set of most influential nodes in online social networks, which is defined as the nodes with the highest k-core value [12]. We employ the *k*-core decomposition method to obtain the cores of different snapshots. The *k*-core decomposition method could be implemented as shown in Fig. 1. Firstly, remove all 1-degree nodes, and then keep pruning these nodes until no more such nodes remaining, the remained nodes form the node set named 2-core. In the similar manner, repeat the pruning process in a similar way for other nodes in the network which have assigned to the corresponding cores(denoted as 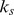). The nodes with the largest *k*-core value is defined as the network core.

The following definitions are given to analyze the evolutional characteristics. The relative growth rate 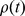 is defined to measure the core growth comparing with the network growth.
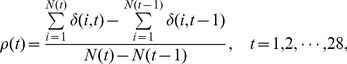
(1)where 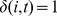 if node *i* exists in the core of the 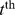 snapshot; Otherwise 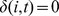. 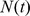 is the number of nodes in the 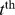 snapshot, and 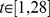. If 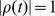, the size change of the cores is the same as that of the network. If 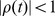, the size change of cores is less in compared with that of the network, otherwise 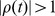.

To give the life span definition, we need to measure the existing times 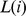 and the continues lifetime 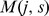. The existing times 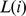 quantifies the number of snapshots that node *i* exists in the 28 cores. The continues lifetime 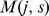 quantifies the number of nodes staying in the cores from the 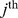 snapshot to the 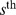 one, which could be defined as follows.
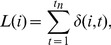
(2)

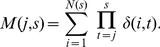
(3)


If 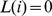, the node *i* never appears in any core, and 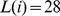 means that the node *i* stays in all 28 cores. 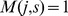 means that there is one node stays in the core from the 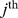 snapshot to the 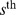 one, and 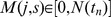. According the above definitions, we can give the distribution 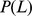 of the existing times 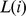 and the distribution 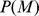 of the nodes who exists in the cores from the 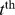 snapshot to the last one.
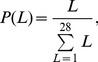
(4)

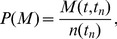
(5)where the 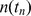 is the core size of the last snapshot. To investigate the connection patterns from the viewpoints of the *k*-core(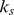) and the degree(*k*), the correlation between the 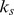 value of the core element and the 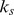 values of its neighbors and corresponding 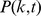 are defined as follows.



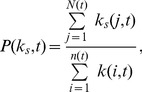
(6)

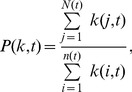
(7)where 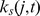 is the 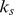 value of node *j* in the 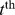 snapshot. 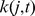 is the degree of node *j* in the 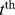 snapshot. The node *j* is one of the core neighbors. 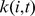 is the degree of the core node *i* in the 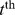 snapshot. The 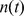 is the core size of the 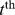 snapshot.

### Additional methods

The properties of the core including assortative coefficient (*r*(*t*)) [23], clustering coefficient (*c*(*t*)) [24], and the average shortest path lengths (*l*(*t*)) are detailed as follows. The assortative coefficient is a measure of the likelihood for nodes which connect to other nodes with similar degrees. A general measure of assortative coefficient is given by [23].
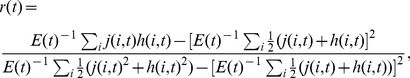
(8)where 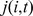, 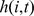 are the degrees of the nodes of the 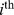 link in the core of the 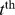 snapshot, for 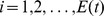. The assortative coefficient value ranges between −1 and 1. By construction, this formula yields 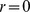 when the amount of assortative mixing is the same as that expected independently at random *i*. A positive assortative coefficient value means that nodes tend to connect to the nodes with similar degree, while a negative assortative coefficient value means that nodes likely connect to nodes with very different degrees from their own.

The clustering coefficient is calculated as follows [24],
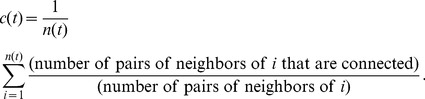
(9)


To understand how the shortest path lengths of the network core change in the evolving process, the average shortest path lengths *l* is used to express as follows.
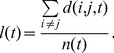
(10)where 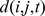 is the shortest path distance between node *i* and *j* in the core of the 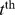 snapshot.

## Results

### The size stability of the core

**Figure 2:**
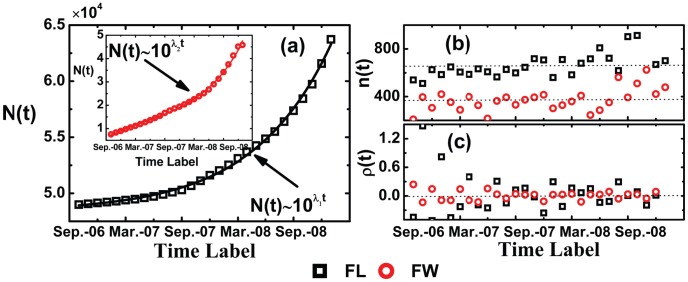
(Color online) Statistical properties of the network size *N(t)* (a) and the corresponding network core sizes *n(t)* (b), as well as the relative growth rate *p(t)* (c). (a) The growing tendency of the size *N(t)* of the FL and FW networks obey an exponential form with exponent λ_1_ = 0.078±0.002 (inset λ_2_ = 0.028±0.002). (b) The core sizes *n(t)* are around 672 and 373. (c) The core relative growth rate *ρ*(*t*) fluctuates around 0.

As shown in Fig. 2(a), the sizes of the FL and FW networks grow exponentially with 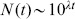, where the parameters 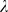 are 0.078 and 0.028 respectively. However, as shown in Fig. 2(b), the core sizes of the FL and FW networks are approximately stable over time. The core relative growth rate 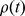 of both the FW and the FL(after 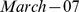) networks fluctuate around zero with time when is as shown in Fig. 2(c). In addition as shown in Tab. 1, we statistics the average core size 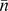 which are equal to 672 and 373 respectively, and the average core relative growth rate value 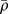 which are equal to 0.040 and 0.009 respectively. That is to say, the size of the core keep stable comparing with the rapid growth of the whole network. Our results suggest that as the Facebook becomes increasingly popular and attracts more and more users, the size of the network grows fast, while the size of core still maintains a stable level.

**Table 1:** The comparisons between the results obtained by the time interval two month (14 snapshots) and the ones obtained by only one month (28 snapshots).

Parameters	λ		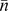		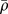	
Time Interval	1	2	1	2	1	2
FL	0.078	0.076	672	673	0.040	0.06
FW	0.028	0.030	373	377	0.009	−0.003

The average value 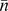 is defined as 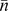 = Σ_*t* = 1_ *n*(*t*)/28; The average value 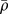 is defined as 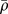 = Σ_*t* = 1_ *ρ*(*t*)/28.

### The evolving topological properties of the core

**Figure 3:**
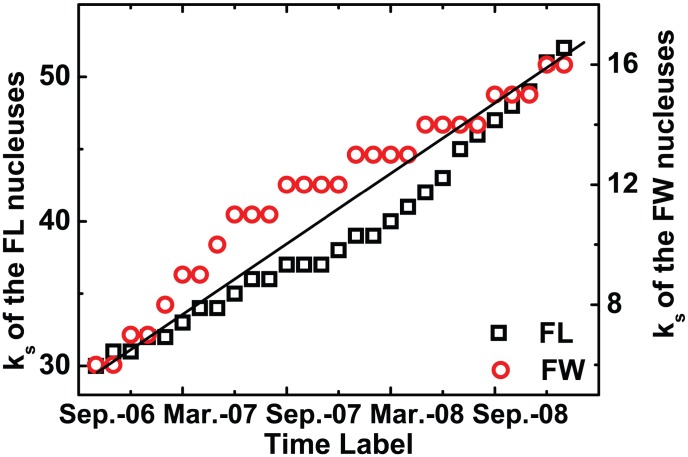
(Color online) The *k_s_* values of each core in the FL and FW networks.

**Figure 4:**
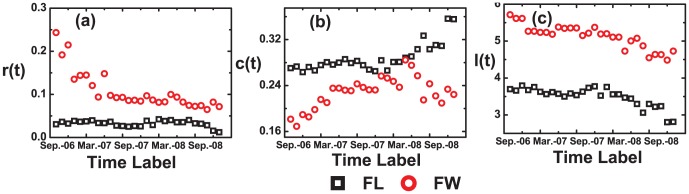
(Color online) The topological properties of the cores. (a) the assortative coefficient *r(t)* is decreasing from 0.25 to 0.1 in the FL network, and fluctuating around 0.05. (b) the average clustering coefficient *c(t)* is increasing slightly. (c) the average shortest path length of the core *l(t)* are decreasing from 3.5 to 3 in the FL network and from 5.7 to 4.5 in the FW network.

From Fig. 3, we could find that the 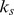 values of cores increase quickly, which indicates that the nodes of cores connect each other more closely. Figure 4 presents the evolving topological properties of the core. In Fig. 4(a), the assortative coefficient 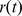 of the FL core is always lower than 0.05, while the 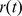 of the FW core keeps decreasing from 0.25 to 0. The results indicate that the users in the Facebook core choose friends to post their comments in walls of their friends independently. They do not care their friends who have be or not be most popular or influential. From Fig. 4(b), one can find that the clustering coefficient 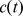 gets larger with time, which indicates the core becomes more interconnected. As shown in Figure 4(c), the shortest path lengths of core gently decreases to 3 in the FL network, and to 4.5 in the FW network as time varying. The decreasing trend also manifests that the core is becoming more interconnected over time.

### The life span of nodes in the core

**Figure 5:**
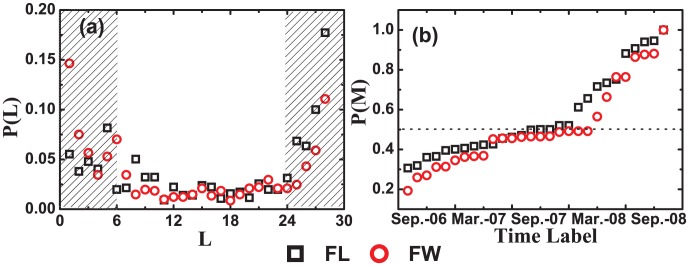
(Color online) The life span of nodes staying in the cores. (a) The distribution P(L) has ‘U’ shaped feature. There are lots of nodes whose existing times are less than 6 or more than 24, and less users whose existing times are larger than 6 and smaller than 24. (b) The distribution P(M) of the nodes who exists in the cores from the *t* ^th^ snapshot to the last one. There are more than 50% nodes belong to the cores for more than one year, 19% nodes always belong to the cores from the first snapshot.

Figure 5(a) shows the distribution of the existing times 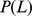, which has a ‘U’ shaped feature. There are lots of nodes whose existing times are less than 6 or more than 24. Meanwhile, we analyze the number of nodes that stay in the core from one snapshot to last snapshot. Figure 5(b) indicates that over 50% nodes stay in the core for more than one year, and 19% nodes always stay in the core from the first snapshot. We suggest that when the Facebook quickly becomes popular and attracts large amounts of users, the most influential and active users will inhabit in the Facebook core for long time.

### The connections between the nodes of core and their neighbors

**Figure 6:**
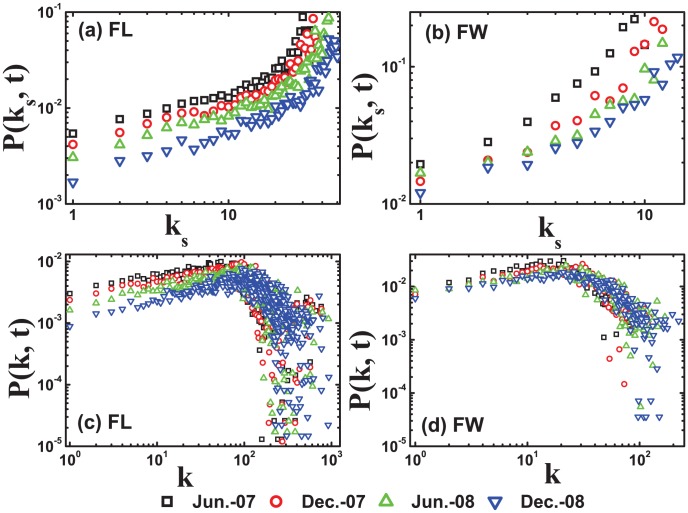
(Color online) The statistics properties of the connections between the core nodes and their neighbors. The time labels are set to Jun. 2007, Dec. 2007, Jun. 2008, and Dec. 2008 which are interval of six months. [(a) and (b)] The correlation between the *k_s_* value of the core element and the *k_s_* values of its neighbors increases with the *k_s_* values in the FL and FW networks. [(c) and (d)] The correlation between the degree *k* of the core element and the degree of its neighbors, from which we could see that nodes in the network have a high probability to connect to core even if they have not largest degrees.

Online social interactions have provided plentiful evidence of their influence for information diffusion. Unfortunately, it is difficult to understand the tendency for individuals who connect to friends with similar tastes or popular preferences [20]. A well-known tendency is that new connections are made preferentially to more popular nodes [21]. Nonetheless, Papadopoulos *et al.* [22] pointed out that the connections should be formed by the trade-off optimization between the popularity and similarity. Here we analyze the core connections from the viewpoints of the 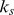 values and degree respectively. As shown in Fig. 6(a) and (b), from which we observe that the correlation between the 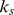 value of the core nodes and the 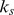 values of its neighbors increases with the 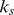 values in the FL and FW networks. We could find that the nodes with larger 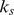 values are more likely to connect to the core. However, as time increasing, the correlation between the 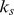 value of the core nodes and the 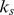 values of its neighbors has fallen with the 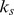 values. Figure 6(c) and (d) show the correlation between the degree k of the core element and the degree of its neighbors, from which we could see that nodes in the network have a high probability to connect to core even if they do not have largest degrees. We could conclude that nodes in the core prefer to connect to nodes with higher 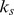 values, rather than the degrees ones.

## Conclusions and Discussions

In this article, we empirically investigate the evolving characteristics of the core of the Facebook. We separate the Facebook-link(FL) and Facebook-wall(FW) networks into 28 snapshots in terms of timestamps, and employ the *k*-core decomposition method to identify the core of each snapshot. The empirical results show the number of users grows exponentially in the evolving process, while the core sizes approximately keep stable levels about 672 and 373 for the FL and FW networks respectively. We also analyze topological properties of the core including the 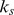 values, assortative coefficient 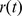, clustering coefficient 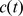 and the average shortest path length 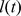 versus time *t*. The 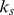 values of cores increase quickly. The assortative coefficient 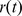 of the FL core is always lower than 0.05, while the 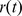 of the FW core keeps decreasing from 0.25 to 0. The clustering coefficient 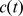 gets larger with time, which indicates the core becomes more interconnected. The shortest path length of core gently decreases from 3.5 to 3 in the FL network and from 5.7 to 4.5 in the FW network. From these topological properties of the core, we could conclude that the users in the core become more interconnected. Furthermore, we analyze the life span of nodes belonging to the core. The distribution of the existing times 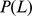 indicates that there are lots of nodes whose existing times are less than 6 or more than 24. Specially the distribution of the continues lifetime 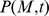 indicates that more than 50% nodes stay in the core for more than one year, and 19% nodes always belong to the core from the first snapshot. We estimated that the most influential users stay in the Facebook core for a long time. Finally, we analyze the connections of individuals in the core. The correlations between the 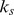 value(*k*) of the core element and the 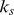
(*k*) values of its neighbors indicate that the users in core prefer to make interactions with network users with higher *k*-core values, regardless of the high degree ones.

**Figure 7:**
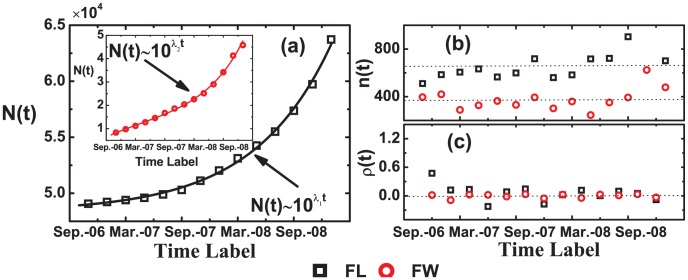
(Color online) Statistical properties of the network size *N(t)* (a) and the corresponding network core sizes *n(t)* (b), as well as the relative growth rate *p(t)* (c). (a) The growing tendency of the size *N*(*t*) of the FL and FW networks obey an exponential form with exponent λ_1_ = 0.076±0.002 (inset λ_2_ = 0.030±0.002). (b) The core sizes *n(t)* are around 672 and 373. (c) The core relative growth rate *p*(*t*) fluctuates around 0.

Our analysis only focused on the evolutional characteristics of the network core in the Facebook, but some additional researches are necessary to complete our findings. First, in this paper, we investigate the evolution properties of the Facebook core with the time interval one month. However, the identification of the network core is affected by the time interval, therefore we also investigated the corresponding results with the time interval two month as shown in the Fig. 7 and Tab. 1, and find that the core size, relative growth rate and other statistical characteristics are robust with the time interval, which suggests that the results obtained in the paper is independent with the time interval. It also should be emphasized that the interactions in online social networks are evolving rapidly, therefore, how to model the temporal relationship between each pair of users and identify the corresponding network core is still an open question for the online social network analysis. Second, although the *k*-core definition of the undirect network core is parameter-free and effective to implement, a lot of online social networks are directed weighted networks which are not suitable for the implementation of the *k*-core decomposition method. To further validate the work presented here, our work will develop a reliable identification of core for directed weighted networks.

In addition, our research could supple some important criteria for modeling the core of the online social networks. In a broader context, our work may be relevant to construct dynamical core model to understand the evolution of the online social network core deeply. User interactions on the online social networks also affect the user behaviors, thus the user behaviors should not just consider the influence of the online societies, but also the influence of offline societies. Specially, the offline social influence could change the user behavior, and then may cause users to leave, which may trigger further leaves of others who lost connections to their friends. This may lead to cascades of users leaving and change the online social network topological structures dramatically. Hence, How to quantify the influence of the offline societies in these online systems can also be an interesting and important open problem.

## References

[1] Centola D (2010) The spread of behavior in an online social network experiment. Science 329: 1194–1197. 20813952 10.1126/science.1185231

[2] Goel S, Watts DJ, Goldstein DG (2012) The structure of online diffusion networks. In Proceedings of the 13th ACM Conference on Electronic Commerce. pp 623–638.

[3] Ugander J, Backstrom L, Marlow C, Kleinberg J (2012) Structural diversity in social contagion. Proc Natl Acad Sci USA 109: 5962–5966. 22474360 10.1073/pnas.1116502109 PMC3341012

[4] Williams AL, Merten MJ (2008) A review of online social networking profiles by adolescents: Implications for future research and intervention. Adolescence 43: 253–274. 18689100

[5] Onnela JP, Reed-Tsochas F (2010) Spontaneous emergence of social influence in online systems. Proc Natl Acad Sci USA 107: 18375–18380. 20937864 10.1073/pnas.0914572107 PMC2972979

[6] Wilson RE, Gosling SD, Graham LT (2012) A review of Facebook research in the social sciences. Perspectives on Psychological Science 7: 203–220. 26168459 10.1177/1745691612442904

[7] Ellison NB (2007) Social network sites: Definition, history, and scholarship. Journal of Computer-Mediated Communication 13: 210–230.

[8] Seidman SB (1983) Network structure and minimum degree. Social networks 5: 269–287.

[9] Carmi S, Havlin S, Kirkpatrick S, Shavitt Y, Shir E (2007) A model of Internet topology using k-shell decomposition. Proc Natl Acad Sci USA 104: 11150–11154. 17586683 10.1073/pnas.0701175104 PMC1896135

[10] Zhang GQ, Zhang GQ, Yang QF, Cheng SQ, Zhou T (2008) Evolution of the Internet and its cores. New J Phys 10: 123027.

[11] Orsini C, Gregori E, Lenzini L, Krioukov D (2013) Evolution of the Internet k-Dense structure. Networking, IEEE/ACM Transactions on 99: 1.

[12] Kitsak M, Gallos LK, Havlin S, Liljeros F, Muchnik L, et al (2010) Identification of influential spreaders in complex networks. Nat Phys 6: 888–893.

[13] Miorandi D, De Pellegrini F (2010) K-shell decomposition for dynamic complex networks. In Modeling and Optimization in Mobile, Ad Hoc and Wireless Networks (WiOpt), 2010 Proceedings of the 8th International Symposium on. pp 488–496.

[14] Ren ZM, Zeng A, Chen DB, Liao H, Liu JG (2014) Iterative resource allocation for ranking spreaders in complex networks. Europhys Lett 106: 48005.

[15] Garas A, Schweitzer F, Havlin S (2012) A k-shell decomposition method for weighted networks. New J Phys 14: 083030.

[16] Viswanath B, Mislove A, Cha M, Gummadi KP (2009) On the evolution of user interaction in facebook. In Proceedings of the 2nd ACM workshop on Online social networks. pp 37–42.

[17] Feige U, Peleg D, Kortsarz G (2001) The dense k-subgraph problem. Algorithmica 29: 410–421.

[18] Palla G, Barabási A L, Vicsek T (2007) Quantifying social group evolution. Nature 446: 664–667. 17410175 10.1038/nature05670

[19] Siganos G, Tauro SL, Faloutsos M (2006) Jellyfish: A conceptual model for the as internet topology. Journal of Communications and Networks 8: 339–350.

[20] Aral S, Muchnik L, Sundararajan A (2009) Distinguishing influence-based contagion from homophily-driven diffusion in dynamic networks. Proc Natl Acad Sci USA 106: 21544–21549. 20007780 10.1073/pnas.0908800106 PMC2799846

[21] Barabási AL, Albert R (1999) Emergence of scaling in random networks. Science 286: 509–512. 10521342 10.1126/science.286.5439.509

[22] Papadopoulos F, Kitsak M, Serrano MA, Boguna M, Krioukov D (2012) Popularity versus similarity in growing networks. Nature 489: 537–540. 22972194 10.1038/nature11459

[23] Newman MEJ (2002) Assortative mixing in networks. Phys Rev Lett 89: 208701. 12443515 10.1103/PhysRevLett.89.208701

[24] Watts DJ, Strogatz SH (1998) Collective dynamics of ‘small-world’ networks. Nature 393: 440–442. 9623998 10.1038/30918

